# Utilizing machine learning and MALDI-TOF MS platform for rapid detection of ceftriaxone-resistant nontyphoidal *Salmonella*

**DOI:** 10.1128/spectrum.00984-26

**Published:** 2026-09-28

**Authors:** Jintao Xia, Jun Ren, Shifu Wang, Mengyu Zhang, Xiaochen Sun, Chao Fang, Shixing Liu, Xiucai Zhang, Yining Zhao, Lijia Wang, Shiqiang Shang, Shengjie Li, Wenjun Cao, Mingming Zhou

**Affiliations:** 1 Department of Laboratory Medicine, Children's Hospital, Zhejiang University School of Medicine, National Clinical Research Center for Children and Adolescents' Health and Diseases 605254, Hangzhou, China; 2 Department of Clinical Laboratory, Eye & ENT Hospital, Fudan University 159395, Shanghai, China; 3 Department of Clinical Microbiology, Children’s Hospital Affiliated to Shandong University (Jinan Children’s Hospital) 576219, Jinan, China; 4 Department of Clinical Laboratory, Wanbei Coal Electric Group General Hospital 645365 , Suzhou, Anhui Province, China; 5 Department of Anesthesiology, The First Affiliated Hospital, Zhejiang University School of Medicine 71069 , Hangzhou, Zhejiang, China; 6 Children’s Hospital, Zhejiang University School of Medicine, National Clinical Research Center for Children and Adolescents' Health and Diseases 605254, Hangzhou, Zhejiang, China; 7 Zhejiang Key Laboratory of Neonatal Diseases, Hangzhou, China; University of Georgia College of Veterinary Medicine, Athens, Georgia, USA

**Keywords:** nontyphoidal *Salmonella*, ceftriaxone, MALDI-TOF MS, machine learning, SHAP

## Abstract

Ceftriaxone (CRO) is a first-line antibiotic for pediatric nontyphoidal *Salmonella* (NTS) infections, but rising resistance threatens public health. Conventional antimicrobial susceptibility testing remains time-consuming, delaying treatment. Although MALDI-TOF MS enables rapid microbial identification, it lacks inherent resistance detection. This study integrates MALDI-TOF MS with machine learning to predict CRO resistance in NTS. Using 472 isolates (1,414 spectra) from three medical centers, we developed and validated a model based on the Light Gradient Boosting Machine (LGB) algorithm. With only seven features, LGB achieved AUCs of 0.94 (training), 0.88 (internal validation), 0.79 (external validation 1), and 0.80 (external validation 2). We further deployed the model as an interactive Streamlit web interface to demonstrate its potential usability and facilitate future laboratory evaluation. This approach provides a proof-of-concept framework for rapid preliminary prediction of ceftriaxone resistance in NTS using routinely generated MALDI-TOF MS spectra. However, further large-scale, prospective, multicenter validation is required before this model can be implemented as a routine clinical susceptibility testing tool.

**IMPORTANCE:**

The increasing prevalence of ceftriaxone-resistant nontyphoidal *Salmonella* in pediatric patients, particularly in cases requiring antimicrobial therapy, highlights the need for faster antimicrobial susceptibility testing. In this study, we developed a machine learning model integrated with MALDI-TOF MS to predict ceftriaxone resistance in NTS and deployed it as a web-based research-use interface. This proof-of-concept approach may support the future development of rapid adjunctive tools for antimicrobial resistance prediction.

## INTRODUCTION

*Salmonella* is a gram-negative bacterium that can be classified into typhoidal, paratyphoid, and nontyphoidal *Salmonella* (NTS) based on serological classification in clinical practice (1). Typhoidal and paratyphoid *Salmonella* typically cause systemic infections, presenting with symptoms of typhoid and paratyphoid fever, and are mostly confined to endemic regions (2). In contrast, NTS primarily causes gastrointestinal infections, such as diarrhea and gastroenteritis (3). This represents a major public health challenge worldwide, as NTS is a leading cause of foodborne illnesses, especially in vulnerable populations like children, who require effective antibiotic treatment (4, 5). Due to the potential severe side effects of fluoroquinolones in children, third-generation cephalosporins, particularly ceftriaxone (CRO), are commonly used for the treatment of severe or high-risk pediatric NTS infections and are also widely used as first-line agents for antimicrobial susceptibility testing in clinical laboratories (6). The overuse of antibiotics and other factors have contributed to the growing issue of antibiotic resistance, which is also evident in NTS, as resistance to third-generation cephalosporins continues to rise. In pediatric patients, the main resistance mechanisms to ceftriaxone in NTS are the production of extended-spectrum β-lactamases (ESBLs), such as *CTX-M* and *TEM* enzymes, as well as AmpC β-lactamases. Our research group previously conducted a study that analyzed NTS isolated from pediatric patients between 2019 and 2020, revealing a resistance rate to ceftriaxone of 25.9% (7). Another study analyzing pediatric NTS infections in Taiwan between 2012 and 2019 found that the resistance rate to CRO increased significantly from 4.1% in 2012 to 14.3% in 2019. Notably, among infants under one year of age, the resistance rate in 2019 reached as high as 25.3% (8). It poses a serious threat to both clinical treatment and public health.

Traditional methods for detecting antibiotic resistance in bacterial pathogens often rely on time-consuming culture and susceptibility testing, which can delay effective treatment and negatively impact patient outcomes (9). As a result, the demand for rapid and accurate diagnostic tools is more critical than ever. Recent advances in mass spectrometry, particularly matrix-assisted laser desorption ionization time-of-flight mass spectrometry (MALDI-TOF MS), have revolutionized bacteria identification and characterization (10, 11). MALDI-TOF MS allows for rapid species-level identification of bacteria, and its application in determining antibiotic resistance patterns is becoming increasingly popular (12, 13). Machine learning techniques can analyze complex data sets, uncover intricate relationships within the data, and make informed decisions (14). Studies have shown that machine learning combined with MALDI-TOF MS enables rapid prediction of antimicrobial resistance, accurately identifying vancomycin resistance in *Enterococcus faecium* and ciprofloxacin and tetracycline resistance in *Campylobacter* spp. (15, 16). However, studies on CRO susceptibility in NTS remain limited.

This study aimed to develop and validate an identification model for automatically distinguishing ceftriaxone-resistant nontyphoidal *Salmonella* (CRNS) using MALDI-TOF MS and machine learning algorithms. Feature selection was conducted using the SHapley Additive exPlanations (SHAP) method, and a user-friendly, web-based model was then constructed to facilitate implementation in clinical laboratories.

## MATERIALS AND METHODS

### Bacterial isolates

**Fig 1:**
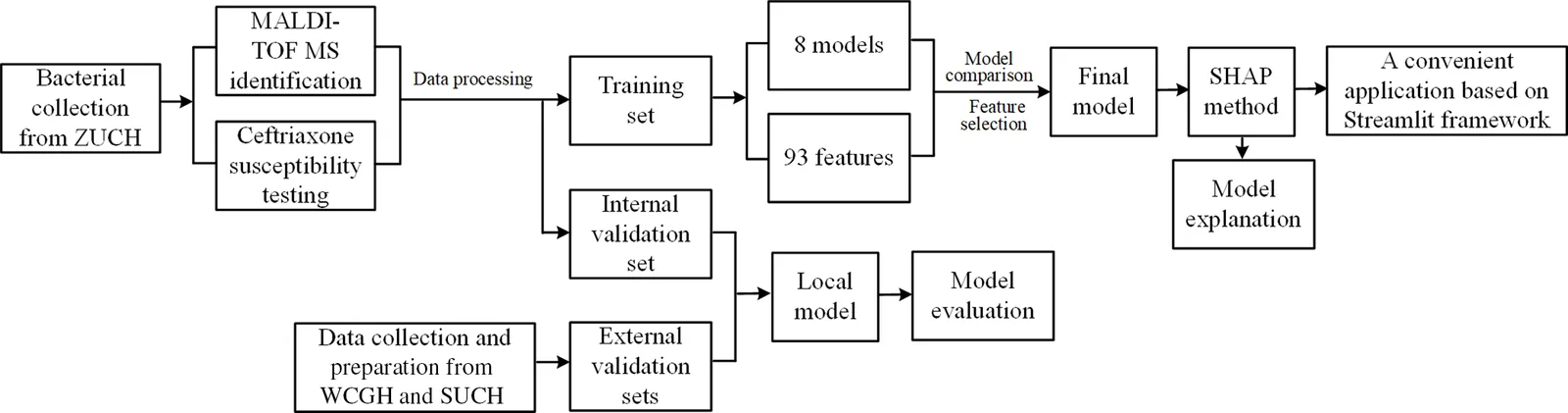
Flow chart of the study design.

Figure 1 shows the detailed flow of the model construction. All isolates were obtained from stool samples of pediatric patients. A total of 472 clinically isolated *Salmonella* strains were included in this study, comprising 387 isolates from Children’s Hospital, Zhejiang University School of Medicine (ZUCH), 25 isolates from the Wanbei Coal-Electricity Group General Hospital (WCGH), and 60 isolates from Children’s Hospital Affiliated to Shandong University (SUCH). All isolates from three centers were cultured overnight at 35°C, analyzed using MALDI-TOF MS at their respective institutions, and subsequently confirmed and serotyped by serological testing (Tian Run, Ningbo, China) to ensure that only nontyphoidal *Salmonella* isolates were included. Salmonella-Shigella agar was selected because it is routinely used in our clinical microbiology laboratories for the primary screening and isolation of *Salmonella* from clinical samples, thereby reflecting the standard diagnostic workflow in our institution.

### Acquisition of protein spectra

The protein extraction procedure for MALDI-TOF MS analysis was performed as previously described (17). Isolate identification was conducted using MALDI-TOF MS in conjunction with the Biotyper system (version 3.1; Bruker Daltonics, Bremen, Germany). A single colony was applied to each target spot, followed by the addition of 1 μL of 70% (vol/vol) formic acid. After air-drying at room temperature, an equal volume of α-cyano-4-hydroxycinnamic acid (HCCA) matrix solution (saturated solution in 50% acetonitrile and 2.5% trifluoroacetic acid) was added to the spot and allowed to dry again. Protein spectra were acquired in the range of 2,000–20,000 Da. Each isolate was spotted in triplicate on the MALDI target plate. To ensure the quality of the data used for machine learning model construction, spectra with identification scores below 2.0 were excluded from further analysis.

### Processing of protein spectra from MALDI-TOF MS

Due to the variations in protein spectra processing software across different MALDI-TOF MS manufacturers, we selected the R packages “MALDIquant (version 1.21)” and “MALDIquantForeign (version 1.2)” for our analysis. This choice streamlined the protein spectra processing steps, making them more convenient and reproducible. Initially, the data were exported in TXT format and imported into R for processing. To stabilize the variance in signal intensities and mitigate the impact of extreme values, a square root transformation (method = “sqrt”) was applied. The Savitzky-Golay filter, with a window size of 90, was then used to smooth the data, reducing noise while retaining the main spectral peaks. Baseline correction was performed using the SNIP method with 100 iterations to remove systematic baseline drift. The spectra were subsequently aligned using the LOWESS method, with a signal-to-noise ratio of 2 and a tolerance of 0.008. Ultimately, 93 protein feature peaks were retained for further analysis. The specific data preprocessing code can be found in the Supplemental materials.

### Antimicrobial susceptibility testing

CRO susceptibility testing was performed for all isolates using the Kirby-Bauer disk diffusion method or minimum inhibitory concentration (MIC) method, in accordance with the Clinical and Laboratory Standards Institute (CLSI) guidelines published annually (18). Ceftriaxone disks (30 μg) were purchased from Oxoid Ltd. (Basingstoke, UK). *Escherichia coli* ATCC 25922 was used as the quality control strain. Briefly, a 0.5 McFarland standard bacterial suspension was prepared from fresh overnight cultures and evenly spread onto Mueller-Hinton agar plates (MHA, Biocell, Zhengzhou, China). Antibiotic disks were then placed on the surface of the inoculated plates, which were incubated overnight at 35°C. Following incubation, the diameter of the inhibition zones was measured, and susceptibility interpretations were made based on CLSI breakpoints (M100, 33rd edition).

### Development and evaluation of models

The batch of 1,161 peak lists (CRNS [234], non-CRNS [927]) was collected from ZUCH. The CRNS (171) and non-CRNS (693) groups were used as the training set, while a batch of 297 peak lists served as the internal validation set. External validation was conducted using 75 peak lists collected from WCGH (CRNS [12], non-CRNS [63]) and 178 peak lists collected from SUCH (CRNS [58], non-CRNS [120]). To avoid data leakage, data set partitioning was performed at the isolate level rather than the spectrum level. Because each isolate was analyzed in triplicate by MALDI-TOF MS, all spectra derived from the same isolate were assigned to the same data set. Thus, replicate spectra from a single isolate were not split across the training, internal validation, or external validation sets. An identification model was constructed based on 93 protein feature peaks. Median imputation was applied to handle missing data, as it preserves the original distribution characteristics without artificially reducing variance; features with more than 10% missing values were excluded. Missing value imputation was performed using pandas 2.2.3, with detailed procedures provided in the Supplemental code. Eight machine learning algorithms were employed to predict CRO susceptibility in NTS, including adaptive boosting (AB), light gradient boosting (LGB), k-nearest neighbors (KNN), logistic regression (LR), random forest (RF), support vector machine (SVM), extreme gradient boosting (XGB), and Gaussian naive Bayes (GNB). The scikit-learn 1.4.2 was used to implement these models, with specific codes available in the Supplemental materials. Model optimization was achieved through a combination of grid search and manual tuning, using the average area under the receiver operating characteristic curve (AUC) from fivefold cross-validation as the evaluation metric for determining optimal hyperparameters. Model performance was assessed based on AUC, sensitivity, specificity, positive predictive value (PPV), negative predictive value (NPV), accuracy, and F1 score. The DeLong nonparametric method was employed to compare AUCs between models and assist in selecting the best-performing model.

### Feature reduction and model determination

Mass spectrometry data often contain numerous complex features, and feature reduction is essential to eliminate noise, reduce model complexity, and enhance interpretability. SHAP, a method based on game theory, was used to quantify the contribution of each feature to the model output. For the binary classification models developed in this study, SHAP provided insights into the influence of individual features on the classification outcome. Based on the ranking of feature importance, the identification model was progressively simplified, reducing from 93 features down to a single feature. As the number of features decreased, the AUC of the selected model gradually declined. The DeLong nonparametric method was used to compare the AUCs between the full feature set and the reduced feature sets. A *P*-value < 0.05 was considered statistically significant, and the previous feature set was selected as the optimal set for building the final model. Using the finalized model, performance was evaluated in the ZUCH internal validation cohort and two independent external validation cohorts from WCGH and SUCH.

### Web implementation of the model

To demonstrate the potential usability of the final identification model and facilitate further laboratory evaluation, we deployed it as a web-based research-use interface using the Streamlit Python framework. In the web interface, users can input the relative intensity values corresponding to each of the seven selected mass spectrometry feature peaks into designated fields. Upon entering all required values, users can click the “Predict” button, and the application calculates and displays the predicted probabilities of ceftriaxone-susceptible and ceftriaxone-resistant outcomes for NTS. The detailed step-by-step protocol is provided in Supplemental materials.

### Statistical analysis

Protein spectra processing was conducted using R (version 4.3.2) and RStudio (2023.03.1+446). Machine learning modeling, as well as the generation of receiver operating characteristic (ROC) and precision‒recall (PR) curves, was performed with Python (version 3.11) and PyCharm (2023.3.5). The DeLong test was used to compare AUC values between different models. The final machine learning model was deployed and visualized using the Streamlit framework. A two-tailed *P*-value < 0.05 was considered statistically significant.

## RESULTS

### Feasibility study

Among the 472 isolates included in this study, all were classified as either susceptible or resistant, and no intermediate isolates were identified according to CLSI interpretive criteria. The distribution of ceftriaxone susceptibility results, determined by either the Kirby–Bauer disk diffusion method or the MIC method, was analyzed, and detailed results are provided in Table S1.

**Fig 2:**
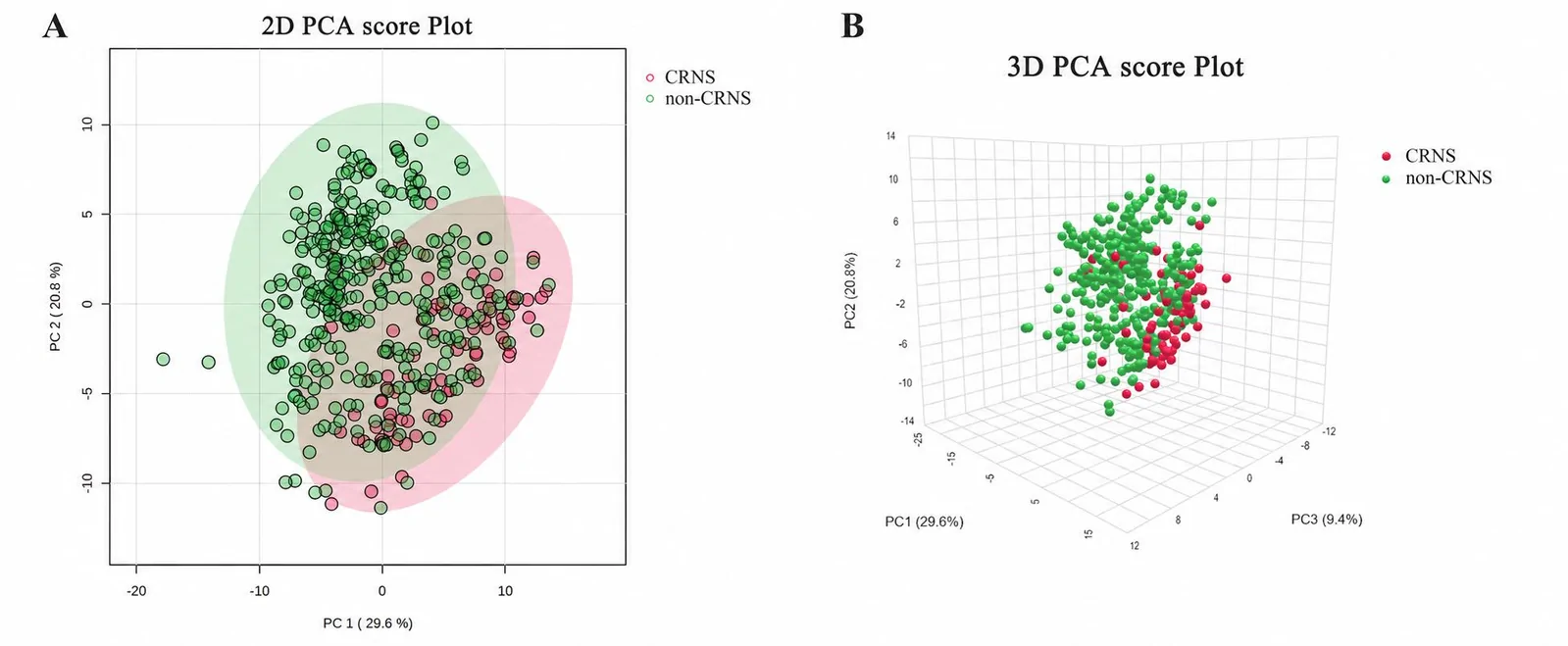
Principal component analysis (PCA) of ceftriaxone-resistant and -susceptible NTS isolates based on MALDI-TOF MS features. (**A**) Two-dimensional PCA score plot showing the distribution of NTS isolates along the first two principal components (PC1 and PC2). (**B**) Synchronized 3D PCA score plot illustrating sample separation along PC1, PC2, and PC3. Each point represents an individual isolate, with colors indicating ceftriaxone susceptibility status (resistant vs susceptible). PCA was conducted in an unsupervised manner to explore intrinsic clustering tendencies between the two groups prior to model construction.

To preliminarily explore whether MALDI-TOF MS-derived features exhibited distributional differences between ceftriaxone-resistant and ceftriaxone-susceptible NTS isolates, an unsupervised principal component analysis (PCA) was performed. As shown in Fig. 2A, the two-dimensional PCA score plot showed a partial separation trend between the two groups, suggesting that the spectral profiles may contain information related to ceftriaxone susceptibility. The synchronized three-dimensional PCA plot (Fig. 2B) provided an additional visualization of the sample distribution across the first three principal components. However, as PCA is an unsupervised exploratory method, these results should not be interpreted as evidence of classification capability. The discriminatory performance of MALDI-TOF MS-derived features was further evaluated using supervised machine learning models and independent validation cohorts.

### Development and comparative evaluation of models

Using the training data set comprising 93 features, eight machine learning models were developed to predict CRO susceptibility (Fig. S1). The precision–recall (PR) curves for all models are presented in Fig. S2. Summary performance metrics, including average AUC, sensitivity, specificity, PPV, NPV, accuracy, and F1 score, are listed in Table 1. The parameters of the eight models are provided in Table S2. To guide model selection, DeLong’s test was employed to compare the AUCs across models, with corresponding *P*-values for pairwise comparisons shown in Table S3. Based on these results, models that were statistically inferior were excluded, and five models—LGB, RF, AB, GNB, and XGB—were retained due to their superior classification performance.

**TABLE 1:** The binary classification performance of machine learning models across different data sets^*a*^

	Models	AUC	Sensitivity	Specificity	PPV	NPV	Accuracy	F1 score
Train set (93 features)	RF	0.92	0.95	0.65	0.92	0.78	0.89	0.93
	AB	0.91	0.92	0.65	0.92	0.66	0.86	0.92
	KNN	0.87	0.93	0.67	0.92	0.73	0.88	0.92
	GNB	0.89	0.78	0.86	0.96	0.50	0.79	0.86
	LR	0.83	1	0	0.80	NA	0.80	0.89
	XGB	0.90	0.95	0.70	0.93	0.79	0.90	0.94
	LGB	0.93	0.94	0.65	0.92	0.74	0.89	0.93
	SVM	0.92	1	0.02	0.80	NA	0.81	0.89
Train set (seven features)	XGB	0.90	0.94	0.67	0.92	0.76	0.89	0.93
	AB	0.89	0.93	0.72	0.93	0.73	0.89	0.93
	LGB	0.94	0.96	0.74	0.94	0.83	0.91	0.95
	GNB	0.89	0.87	0.72	0.93	0.60	0.84	0.90
	RF	0.91	0.94	0.54	0.90	0.69	0.86	0.91
Internal validation set (seven features)	XGB	0.84	0.86	0.67	0.91	0.56	0.82	0.88
	AB	0.83	0.86	0.71	0.92	0.58	0.83	0.89
	LGB	0.88	0.91	0.81	0.95	0.71	0.89	0.93
	GNB	0.76	0.71	0.81	0.93	0.43	0.73	0.80
	RF	0.84	0.90	0.52	0.88	0.58	0.82	0.89
WCGH (seven features)	XGB	0.86	0.95	0.75	0.95	0.75	0.92	0.95
	AB	0.78	0.95	0.50	0.91	0.67	0.88	0.93
	LGB	0.79	0.95	0.75	0.95	0.75	0.92	0.95
	GNB	0.80	0.95	0.50	0.91	0.67	0.88	0.93
	RF	0.85	1	0.50	0.91	1	0.92	0.95
SUCH (seven features)	XGB	0.68	0.82	0.40	0.74	0.51	0.69	0.78
	AB	0.73	0.64	0.68	0.81	0.47	0.65	0.71
	LGB	0.80	0.95	0.66	0.85	0.86	0.85	0.90
	GNB	0.64	0.83	0.33	0.72	0.48	0.67	0.77
	RF	0.72	0.83	0.53	0.79	0.60	0.74	0.81

^*a*^ The evaluation metrics are the averages calculated based on the prediction results of ceftriaxone susceptibility in NTS. PPV, positive predictive value; NPV, negative predictive value. The prevalence of ceftriaxone resistance was 20.16% in the ZUCH group, 16% in the WCGH group, and 33.3% in the SUCH group.

### Feature selection and final model optimization

**Fig 3:**
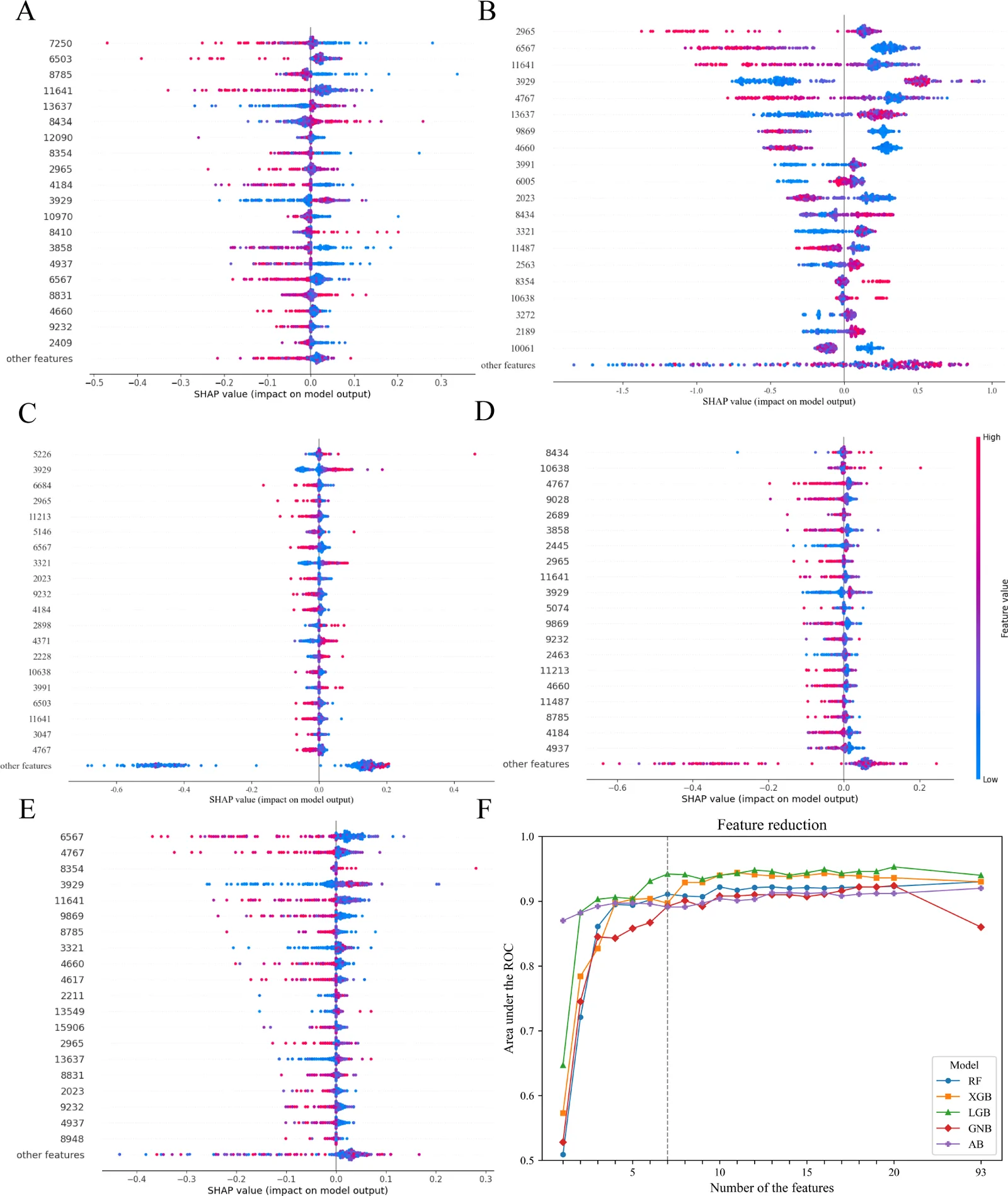
SHAP summary plots and feature selection. SHAP summary plots showing the top 20 features in five ML models for predicting ceftriaxone susceptibility in NTS. (**A**) AB, (**B**) LGB, (**C**) GNB, (**D**) RF, and (**E**) XGB. SHAP values indicate each feature’s contribution to the predicted outcome. Red dots indicate high feature values; blue dots indicate low values. The vertical dispersion of dots shows feature density across samples. (**F**) AUCs of the RF, AB, LGB, GNB, and XGB models with various numbers of features.

Among the five machine learning models, the LGB model demonstrated the highest predictive performance for CRO susceptibility (AUC = 0.93), followed by the RF model (AUC = 0.92) and the AB model (AUC = 0.91). The SHAP summary dot plots highlighting the top 20 contributing features for the five best-performing models are shown in Fig. 3A through E.

During the feature reduction process based on feature importance, the AUC performance of five models was evaluated. Both the LGB and XGB models maintained strong discriminative ability despite the reduction in features, highlighting their stability and robustness in the feature selection process (Fig. 3F). For the LGB model, reducing the number of features from 93 to 6 led to a statistically significant decrease in AUC (0.94 vs 0.9309, *P* = 0.03). Consequently, the model with seven features was identified as the optimal model, as its performance was comparable to the full-feature model (AUC: 0.94 vs 0.9420, *P* = 0.62). Moreover, the 7-feature model provided a greater net clinical benefit and a higher threshold probability compared with the 93-feature model. For the XGB model, reducing the features from 93 to 7 resulted in a significant decline in AUC (0.93 vs 0.8973, *P* < 0.01). Notably, at seven features, the LGB model (AUC: 0.94) outperformed the XGB model (AUC: 0.93). Detailed results are presented in Table S4. Therefore, we selected the 7-feature LGB model—comprising the 2,965, 6,567, 11,641, 3,929, 4,767, 13,637, and 9,869 m/z peaks—as the final model for further analysis.

### Evaluation of the final LGB model in the training set

**Fig 4:**
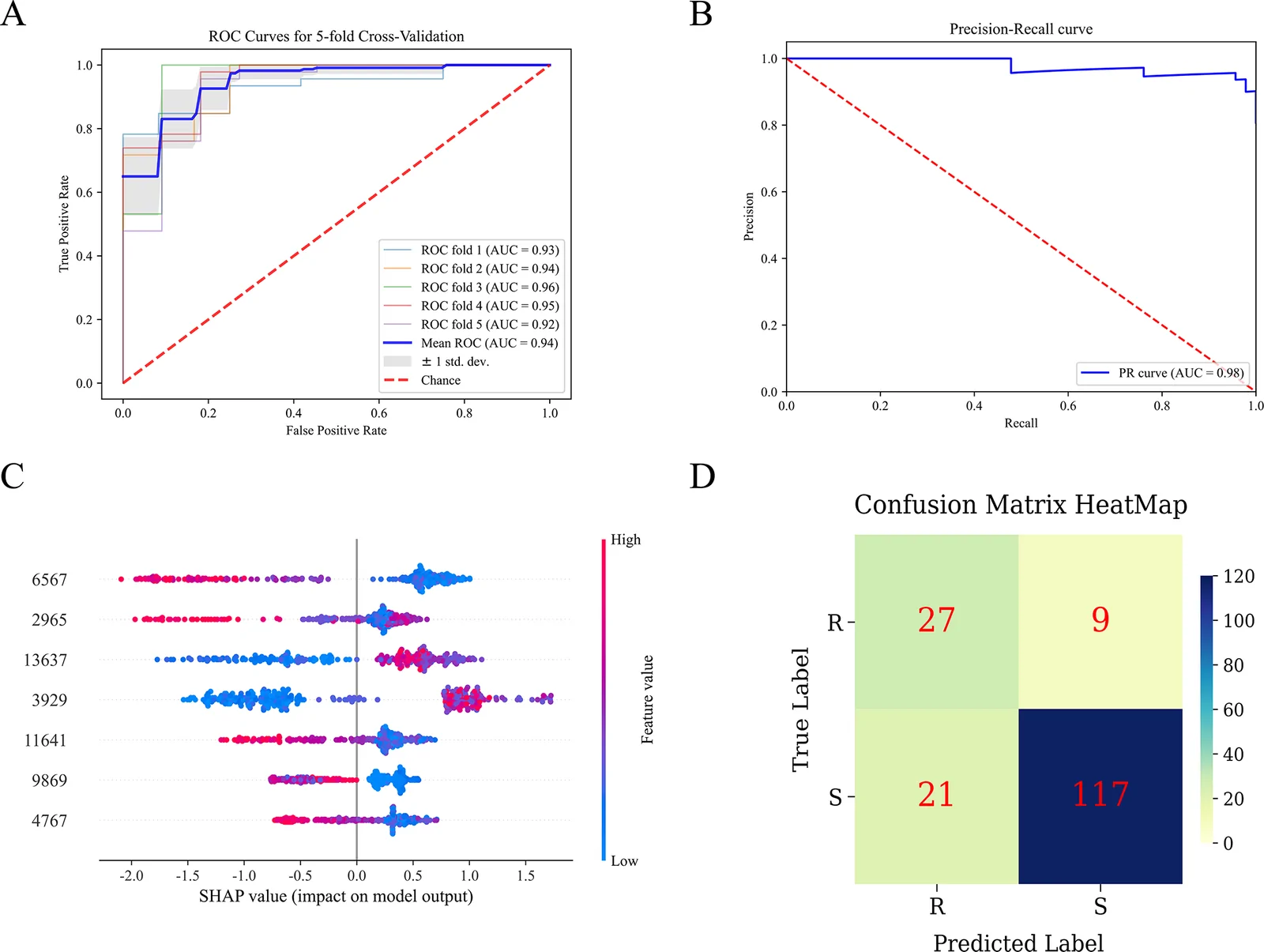
Evaluation of the final LGB model in the training set. (**A**) ROC curve, (**B**) PR curve, (**C**) SHAP summary plot of the final model, and (**D**) confusion matrix heatmap for predicting ceftriaxone susceptibility in NTS. The SHAP plot shows feature importance, with dot color representing actual feature values (red = high, blue = low). The confusion matrix heatmap indicates classification performance. R, resistant; S, susceptible.

To assess the performance of the final LGB model in predicting CRO susceptibility in NTS, we evaluated it on the training set using multiple metrics. ROC curve demonstrated robust classification performance, with a high AUC, indicating good sensitivity and specificity (Fig. 4A). For comparison, the final 7-feature LGB model achieved an AUC of 0.94, with a sensitivity of 0.96, specificity of 0.74, PPV of 0.94, NPV of 0.83, accuracy of 0.91, and F1 score of 0.95 for CRO susceptibility prediction (Table 1). Similarly, the PR curve showed favorable precision across a wide range of recall values, further supporting the model’s effectiveness in handling class imbalance (Fig. 4B).

As shown in SHAP summary plot (Fig. 4C), the contributions of the feature to the model were evaluated using the average SHAP values and exhibited in descending order.

The confusion matrix heatmap visualizes the overall classification performance (Fig. 4D). The majority of samples were correctly classified, with relatively low numbers of false positives and false negatives, supporting the model’s reliability in predicting CRO susceptibility in NTS strains.

The ROC curves, PR curves, and confusion matrix heatmaps from the training set for the 7-feature AB, GNB, RF, and XGB models are shown in Fig. S3 to S6.

### Model interpretation using SHAP dependence and waterfall plots

To further interpret the predictions made by the final LGB model, we visualized SHAP dependence and waterfall plots (Fig. S7). The SHAP dependence plots for the seven most influential features (Fig. S7A through G) revealed how each feature’s value impacted the model’s output on a per-sample basis. In these plots, each dot represents one isolate in the data set; the x-axis shows the raw feature value, while the y-axis represents the corresponding SHAP value. A positive SHAP value indicates a higher likelihood of sensitivity, whereas a negative value suggests resistance. These plots also revealed potential thresholds or nonlinear relationships between certain features and the prediction outcome.

To better illustrate how the model makes predictions at the individual level, SHAP waterfall plots were generated for two representative NTS isolates (Fig. S7H and I). In these plots, each bar represents the contribution of a specific feature to the final predicted risk. Red bars indicate features that push the prediction toward CRO susceptibility, while blue bars represent features that drive the prediction toward resistance. These visualizations highlight how the combined effect of multiple features leads to the final model decision, providing case-level interpretability and helping identify key contributors in resistant versus susceptible isolates.

### Internal and external evaluation of the final model

In the internal validation set, the final model achieved an AUC of 0.88, closely matching its performance in the training set (ΔAUC = 0.06). Similarly, in the external validation sets, the model yielded an AUC of 0.79 and 0.80, with only a modest decline compared to the training set (ΔAUC = 0.15 and 0.14). These findings suggest that the final model maintained robust predictive performance across both internal and external validation cohorts.

**Fig 5:**
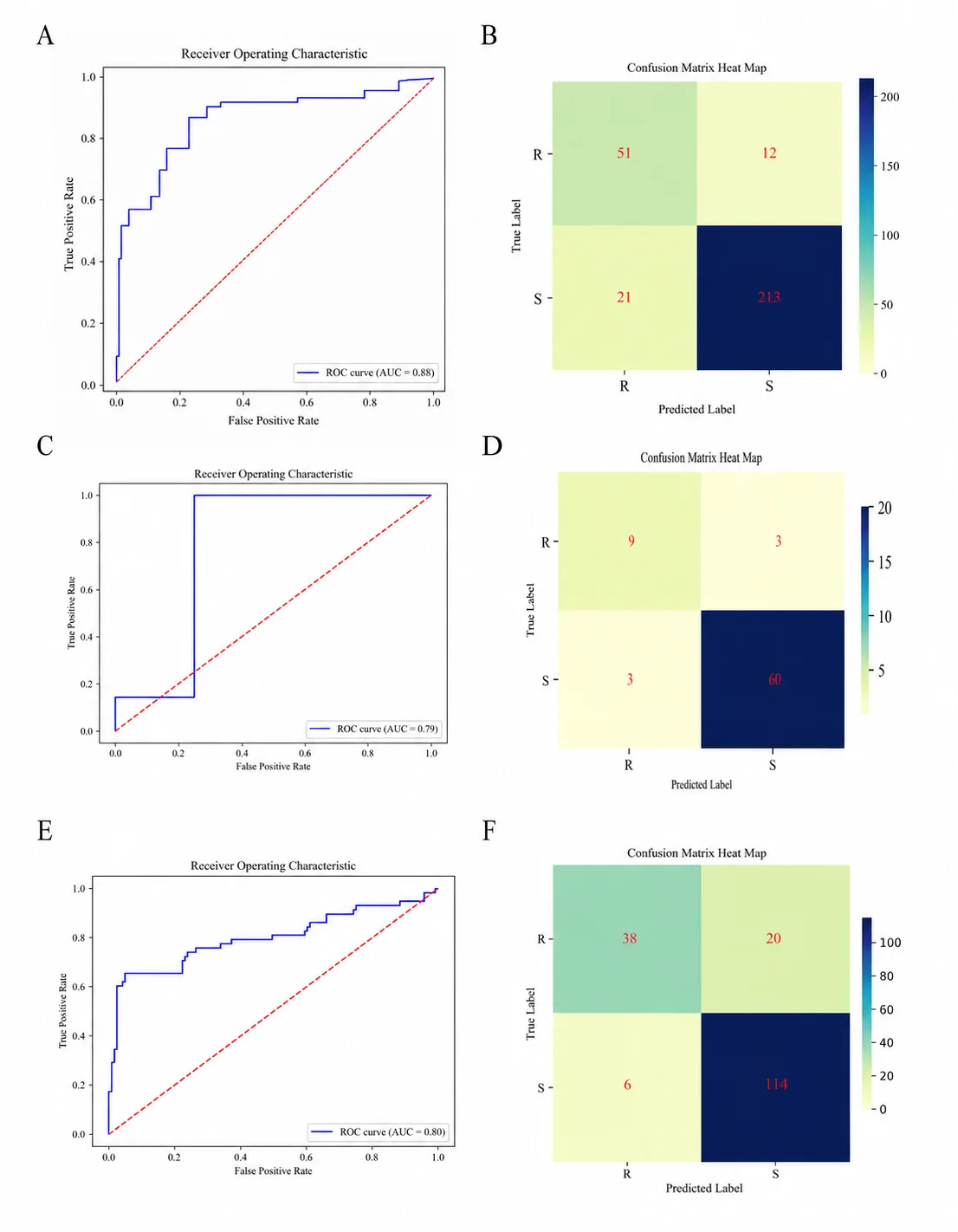
Evaluation of the final LGB model in validation cohorts. (**A and B**) ROC curve and confusion matrix heatmap in the internal validation set. (**C and D**) ROC curve and confusion matrix heatmap in the WCGH set. (**E and F**) ROC curve and confusion matrix heatmap in the SUCH set. The results indicate strong discriminative power and reliable classification performance for predicting ceftriaxone susceptibility. R, resistant; S, susceptible.

The ROC curves and confusion matrix heatmaps for the internal and external validation sets are shown in Fig. 5A through F, while the corresponding PR curve is presented in Fig. S8. The ROC curves, PR curves, and confusion matrix heatmaps for AB, GNB, RF, and XGB across the internal and external validation sets are provided in Fig. S3 to S6. These results collectively demonstrate that the LGB model outperformed the other four models. Furthermore, the average AUC, sensitivity, specificity, PPV, NPV, accuracy, and F1 score for the five models—based on the seven selected features evaluated across the training, internal validation, and external validation sets—are summarized in Table 1.

The decision curve analysis (DCA) demonstrated that the LGB model consistently provided a higher net benefit than the “treat-all” and “treat-none” strategies across a wide range of threshold probabilities in the training set, internal validation set, and external validation sets, providing additional evidence of the model’s performance and its applicability to clinical decision-making (Fig. S9).

The categorical agreement (CA) between the predicted and reference susceptibility results was 84.0%. The very major error (VME) rate was 12.7%, and the major error (ME) rate was 17.1%.

### Visualization of the prediction model

To facilitate practical application of the developed model, we deployed the final identification model as a web-based application using Streamlit, as illustrated in Fig. S10. Users can input the intensity values of the required features, and the application automatically predicts the CRO susceptibility of NTS along with the corresponding prediction probabilities. For example, for the input sample shown in Fig. S10, the model predicted resistance with a probability of 93.7%. The web application is publicly accessible at the following URL: https://shamenny.streamlit.app/. Please note that the web app may occasionally enter a “sleep” mode; if this occurs, allow approximately 30 s for the application to reactivate automatically.

The current version of the tool is intended only for preliminary predictions based on spectra generated under the same conditions as those used in this study. It should not be regarded as a standalone routine diagnostic system or a replacement for conventional antimicrobial susceptibility testing.

## DISCUSSION

With the rising detection rate of NTS in pediatric populations, the traditional empirical use of ceftriaxone has become increasingly challenging (7). Although antibiotics are not always recommended for uncomplicated gastroenteritis and their use may vary depending on clinical guidelines and disease severity, rapid detection of ceftriaxone resistance remains clinically important for severe or high-risk infections requiring antimicrobial therapy. In the absence of antimicrobial susceptibility test (AST) results, clinicians face significant difficulty in making informed treatment decisions. However, conventional AST methods typically require 18–24 h of bacterial culture and analysis, which fails to meet the urgent clinical demand for rapid and accurate diagnosis (9). MALDI-TOF MS has been widely adopted in clinical microbiology laboratories for its rapid, efficient, and cost-effective bacterial identification at the species level (19). Nevertheless, the vast amount of spectral data contained in MALDI-TOF MS output is currently underutilized and primarily limited to species identification. Recent studies have demonstrated that mass spectra can be further exploited for advanced analyses, such as bacterial subtyping and antibiotic resistance prediction, highlighting the broad research and application potential of MALDI-TOF MS (15, 20).

In this study, we developed a proof-of-concept machine learning framework using MALDI-TOF MS-derived protein spectra to predict ceftriaxone susceptibility in NTS. The optimized LGB model, based on seven selected spectral features, showed promising performance in the training, internal validation, and external validation cohorts, supporting the feasibility of extracting resistance-related information from routinely generated MALDI-TOF MS spectra. Nevertheless, the current study should be interpreted as an exploratory model development and validation study rather than as a clinically validated susceptibility testing tool. Therefore, its predictions should be considered preliminary and should be confirmed by conventional antimicrobial susceptibility testing before clinical use. Although this study focuses on ceftriaxone-resistant *Salmonella* in children, the method developed can also be applied to adult isolates if similar resistance issues arise.

Machine learning (ML) is an advanced tool for processing high-dimensional and complex biological data, with broad applications in the analysis of intricate variable associations (21, 22). Among the eight ML models evaluated in this study, the LGB model demonstrated the best overall performance, characterized by a substantial net benefit and high stability across feature-reduction thresholds. As an efficient gradient-boosting tree-based algorithm, LGB offers strong generalization capabilities and rapid training speed, making it particularly well-suited for high-dimensional data sets and imbalanced classification tasks (23, 24). By iteratively minimizing the loss function during model construction, LGB effectively captures nonlinear relationships among features. Given the large number and complexity of protein peaks in MALDI-TOF MS data, rational feature selection is essential. An excessive number of redundant features may lead to model overfitting and reduce clinical interpretability. Therefore, we adopted the SHAP approach—based on game theory—to evaluate feature importance and identify the peaks with the greatest contribution to model output (25). During the process of feature reduction, the LGB model consistently maintained high discriminative performance with a minimal number of features, highlighting its structural advantages and robustness. Due to differences in model architecture and feature selection strategies, feature importance rankings varied across ML models. In this study, we constructed a final LGB model based on seven features. This model achieved high predictive performance while enhancing interpretability and practicality, thereby providing a feasible and efficient approach for rapidly and accurately predicting ceftriaxone susceptibility in NTS. Compared with previous studies, our work offers several key advancements: (i) detailed, reproducible procedures and code for MALDI-TOF MS data preprocessing and machine learning; (ii) incorporation of SHAP for model interpretation to enhance transparency; and (iii) development of a practical framework for deploying predictive models via a web-based platform. Together, these innovations significantly improve the usability, interpretability, and clinical applicability of the proposed approach.

However, there are some limitations in this study. First, all MALDI-TOF MS spectra in this study were generated using the Bruker MALDI-TOF MS platform. Therefore, the current model should be considered Bruker platform specific and may not be directly transferable to other MALDI-TOF MS systems. Differences in instrument hardware, calibration procedures, acquisition parameters, manufacturer-specific data formats, and spectral preprocessing pipelines may influence peak detection and relative intensity values, thereby affecting model performance. Although the present results support the feasibility of using Bruker-derived MALDI-TOF MS spectra combined with machine learning to predict ceftriaxone resistance in NTS, additional validation using other commonly used platforms, such as BioMérieux, is required. Future studies should evaluate whether platform-specific recalibration, harmonized preprocessing, or model retraining is needed to enable reliable cross-platform application. Second, the MALDI-TOF MS platform detects strain-specific spectral features rather than directly identifying the underlying resistance mechanisms. While the model does not distinguish between different resistance mechanisms, we have demonstrated that the spectral features selected for our machine learning model are strongly associated with known resistance profiles. These features were validated by comparison with traditional susceptibility testing, ensuring that the selected features accurately reflect the presence of resistance. Although this approach has shown promising results for the rapid detection of ceftriaxone-resistant NTS, further studies are needed to evaluate the performance of this method in longitudinal settings and across a wider range of environmental conditions to assess its robustness and broad applicability. Third, while MALDI-TOF MS is now widely used, variability in sample preparation and instrument configuration could influence spectral profiles and model performance in other laboratories. Fourth, only Salmonella-Shigella agar was evaluated in this study. This medium was selected because it is routinely used in our clinical microbiology laboratory for the primary screening and isolation of *Salmonella* from stool samples, thereby reflecting our standard diagnostic workflow. However, culture media may influence bacterial protein expression, growth status, and MALDI-TOF MS spectral profiles, which could affect peak detection, relative intensity values, and model performance. Therefore, the current model should be considered applicable only to spectra generated from isolates grown on Salmonella-Shigella agar. Additional validation using other commonly used selective media, such as Hektoen Enteric agar and Xylose Lysine Deoxycholate agar, is required before applying this model to isolates cultured under different media conditions. Fifth, no ceftriaxone-intermediate isolates were identified among the clinically collected NTS isolates according to CLSI interpretive criteria; therefore, the present model was developed as a binary classifier for ceftriaxone-susceptible and ceftriaxone-resistant isolates. The absence of intermediate isolates limits the evaluation of model performance for strains near clinical breakpoints. Future studies including intermediate isolates, if available through expanded multicenter surveillance, are needed to determine whether the model can reliably classify borderline susceptibility phenotypes or whether a separate classification strategy is required. Sixth, the current model was developed and validated only using MALDI-TOF MS spectra with identification scores ≥ 2.0. Therefore, its performance for lower-quality spectra has not been evaluated, and spectra with identification scores < 2.0 should not be used for prediction without repeat acquisition or further validation.

In conclusion, we developed an interpretable LGB model for preliminary prediction of ceftriaxone susceptibility in NTS based on Bruker MALDI-TOF MS-derived spectral features. The final model demonstrated promising performance in the current validation cohorts, supporting the feasibility of this proof-of-concept approach. However, the model should not be considered a clinically validated susceptibility testing tool at this stage. Further large-scale, prospective, multicenter, and multiplatform validation is required before it can be considered for routine clinical implementation.

## Data Availability

The raw MALDI-TOF MS spectra and corresponding metadata generated in this study have been deposited in Zenodo and are available under https://doi.org/10.5281/zenodo.22028741. The analysis code associated with this article is available on GitHub (https://github.com/fudanRenjun/shamenNY.git).

## SUPPLEMENTAL MATERIAL

Supplemental materials spectrum.00984-26-s0001.pdf Supplemental figures, tables, and code.

Table S1 spectrum.00984-26-s0002.xlsx Distribution of ceftriaxone susceptibility results.

ASM does not own the copyrights to Supplemental Material that may be linked to, or accessed through, an article. The authors have granted ASM a non-exclusive, world-wide license to publish the Supplemental Material files. Please contact the corresponding author directly for reuse.
