# Supplementary material for: Utilizing machine learning and MALDI-TOF MS platform for rapid detection of ceftriaxone-resistant nontyphoidal Salmonella
Source: Microbiol Spectr. 2026 Sep 28;14(11):e00984-26. doi: 10.1128/spectrum.00984-26 (PMC13729124; doi:10.1128/spectrum.00984-26)
Supplement: Supplementary file 1 [file spectrum.00984-26-s0001.pdf]

Supplementary Material

1. Table S1: Distribution of ceftriaxone susceptibility results for all isolates in this study, determined by either the Kirby–Bauer disk diffusion method or the minimum inhibitory concentration method.

2.Table S2: List of parameters for eight models.

3.Table S3: The differences in AUC among the 8 models were compared using Delong's nonparametric method.

4.Table S4: The performance of XGB, GNB, LGB, AB and RF models with varying numbers of features for identifying CRO susceptibility in NTS.

5.Figure S1: ROC curves of eight machine learning models for identifying ceftriaxone susceptibility: (A) AB, (B) KNN, (C) LGB, (D) LR, (E) GNB, (F) RF, (G) SVM, and (H) XGB.

6.Figure S2: PR curves for predicting CRO susceptibility using eight machine learning models. (A) AB model. (B) KNN model. (C) LGB model. (D) LR model. (E) GNB model. (F) RF model. (G) SVM model. (H) XGB model.

7.Figure S3: The ROC curves, PR curves and confusion matrix heat maps of the AB model with 7 features on the (A, E, I) training set, (B, F, J) internal validation set, external (C, G, K) WCGH set and (D, H, L) SUCH set.

8.Figure S4: The ROC curves, PR curves and confusion matrix heat maps of the GNB model with 7 features on the (A, E, I) training set, (B, F, J) internal validation set, external (C, G, K) WCGH set and (D, H, L) SUCH set.

9.Figure S5: The ROC curves, PR curves and confusion matrix heat maps of the RF model with 7 features on the (A, E, I) training set, (B, F, J) internal validation set, external (C, G, K) WCGH set and (D, H, L) SUCH set.

10.Figure S6: The ROC curves, PR curves and confusion matrix heat maps of the XGB model with 7 features on the (A, E, I) training set, (B, F, J) internal validation set, external (C, G, K) WCGH set and (D, H, L) SUCH set.

11.Figure S7： SHAP dependence plots for the seven most influential features (A–G) and waterfall plots for two representative isolates (H–I) illustrating feature contributions to ceftriaxone resistance prediction.

12.Figure S8: The PR curves of the final LGB model with 7 features on the internal and external validation sets. (A) Internal validation set. (B) External validation 1 (WCGH). (C) External validation 2 (SUCH).

13.Figure S9: Decision curve analysis (DCA) of the LGB model in three datasets. (A) Training set. (B) Internal validation set. (C) External validation 1 (WCGH). (D) External validation 2 (SUCH). The model demonstrated consistently higher net benefit across a range of threshold probabilities compared to the "treat-all" and "treat-none" strategies in all three datasets.

14.Figure S10: User-friendly web application for ceftriaxone susceptibility prediction based on the final seven-feature LGB model, showing (A) input interface and (B) results interface.

15.Code: Data processing of MALDI-TOF MS protein spectra— Page 18

16. Supplementary Protocol: Step-by-step workflow for using the MALDI-TOF MS–based Streamlit prediction tool.

Table S2: List of parameters for eight models.

	Parameter	Settings
AB	learning_rate	0.2
	n_estimators	100
	algorithm	SAMME.R
LGB	max_depth	10
	learning_rate	0.11
	n_estimators	100
	random_state	42
KNN	n_neighbors	5
LR	max_iter	1000
	random_state	42
RF	max_depth	50
	n_estimators	10
	random_state	42
SVM	kernel	rbf
	C	1.0
	probability	True
	random_state	42
XGB	gamma	5.271
	learning_rate	0.0436
	max_depth	8
	subsample	0.7012
GNB	var_smoothing	1e-07

Table S3: The differences in AUC among the 8 models were compared using Delong's nonparametric method.

Model1	AUC1	Model2	AUC2	P-value
XGB	0.90	RF	0.92	0.40
XGB	0.90	KNN	0.87	0.26
XGB	0.90	AB	0.91	0.68
XGB	0.90	SVM	0.92	0.40
XGB	0.90	LGB	0.93	0.20
XGB	0.90	GNB	0.89	0.70
XGB	0.90	LR	0.83	0.01
RF	0.92	KNN	0.87	0.05
RF	0.92	AB	0.91	0.67
RF	0.92	SVM	0.92	1
RF	0.92	LGB	0.93	0.65
RF	0.92	GNB	0.89	0.22
RF	0.92	LR	0.83	< 0.01
KNN	0.87	AB	0.91	0.12
KNN	0.87	SVM	0.92	0.05
KNN	0.87	LGB	0.93	0.02
KNN	0.87	GNB	0.89	0.46
KNN	0.87	LR	0.83	0.18
AB	0.91	SVM	0.92	0.67
AB	0.91	LGB	0.93	0.38
AB	0.91	GNB	0.89	0.42
AB	0.91	LR	0.83	< 0.01
SVM	0.92	LGB	0.93	0.65
SVM	0.92	GNB	0.89	0.22
SVM	0.92	LR	0.83	< 0.01
LGB	0.93	GNB	0.89	0.09
LGB	0.93	LR	0.83	< 0.01
GNB	0.89	LR	0.83	0.04

Table S4: The performance of XGB, GNB, LGB, AB and RF models with varying numbers of features for identifying CRO susceptibility in NTS.

	Number of features	AUC	Number of features	AUC	P-value
XGB	93	0.93	1	0.5734	<0.01
	93	0.93	2	0.7836	<0.01
	93	0.93	3	0.8274	<0.01
	93	0.93	4	0.8975	<0.01
	93	0.93	5	0.9030	<0.01
	93	0.93	6	0.9041	<0.01
	93	0.93	7	0.8973	<0.01
	93	0.93	8	0.9288	<0.01
	93	0.93	9	0.9290	<0.01
	93	0.93	10	0.9396	<0.01
	93	0.93	11	0.9436	<0.01
	93	0.93	12	0.9412	<0.01
	93	0.93	13	0.9391	0.03
	93	0.93	14	0.9382	0.05
	93	0.93	15	0.9403	0.01
	93	0.93	16	0.9434	<0.01
	93	0.93	17	0.9402	0.01
	93	0.93	18	0.9388	0.04
	93	0.93	19	0.9360	0.15
	93	0.93	20	0.9360	0.15
GNB	93	0.86	1	0.5283	<0.01
	93	0.86	2	0.7453	<0.01
	93	0.86	3	0.8449	0.01
	93	0.86	4	0.8425	<0.01
	93	0.86	5	0.8583	0.77
	93	0.86	6	0.8671	0.22
	93	0.86	7	0.8916	<0.01
	93	0.86	8	0.9008	<0.01
	93	0.86	9	0.8922	<0.01
	93	0.86	10	0.9080	<0.01
	93	0.86	11	0.9084	<0.01
	93	0.86	12	0.9105	<0.01
	93	0.86	13	0.9100	<0.01
	93	0.86	14	0.9100	<0.01
	93	0.86	15	0.9074	<0.01
	93	0.86	16	0.9105	<0.01
	93	0.86	17	0.9163	<0.01
	93	0.86	18	0.9218	<0.01
	93	0.86	19	0.9221	<0.01
	93	0.86	20	0.9235	<0.01

LGB	93	0.94	1	0.6468	<0.01
	93	0.94	2	0.8833	<0.01
	93	0.94	3	0.9029	<0.01
	93	0.94	4	0.9056	<0.01
	93	0.94	5	0.9043	<0.01
	93	0.94	6	0.9309	0.03
	93	0.94	7	0.9420	0.62
	93	0.94	8	0.9412	0.77
	93	0.94	9	0.9342	0.16
	93	0.94	10	0.9402	0.96
	93	0.94	11	0.9429	0.47
	93	0.94	12	0.9479	0.04
	93	0.94	13	0.9465	0.10
	93	0.94	14	0.9395	0.90
	93	0.94	15	0.9443	0.27
	93	0.94	16	0.9489	0.02
	93	0.94	17	0.9428	0.48
	93	0.94	18	0.9460	0.12
	93	0.94	19	0.9459	0.13
	93	0.94	20	0.9527	<0.01
AB	93	0.92	1	0.8703	<0.01
	93	0.92	2	0.8822	<0.01
	93	0.92	3	0.8921	<0.01
	93	0.92	4	0.8973	<0.01
	93	0.92	5	0.8966	<0.01
	93	0.92	6	0.8958	<0.01
	93	0.92	7	0.8912	<0.01
	93	0.92	8	0.8914	<0.01
	93	0.92	9	0.8970	<0.01
	93	0.92	10	0.9040	<0.01
	93	0.92	11	0.9014	<0.01
	93	0.92	12	0.9034	<0.01
	93	0.92	13	0.9130	0.13
	93	0.92	14	0.9131	0.14
	93	0.92	15	0.9119	0.08
	93	0.92	16	0.9129	0.13
	93	0.92	17	0.9078	<0.01
	93	0.92	18	0.9107	0.05
	93	0.92	19	0.9117	0.07
	93	0.92	20	0.9117	0.07
RF	93	0.93	1	0.5090	<0.01
	93	0.93	2	0.7208	<0.01
	93	0.93	3	0.8615	<0.01
	93	0.93	4	0.8953	<0.01

93	0.93	5	0.8941	<0.01
93	0.93	6	0.9023	<0.01
93	0.93	7	0.9107	<0.01
93	0.93	8	0.9081	<0.01
93	0.93	9	0.9074	<0.01
93	0.93	10	0.9222	0.08
93	0.93	11	0.9168	<0.01
93	0.93	12	0.9206	0.03
93	0.93	13	0.9217	0.06
93	0.93	14	0.9196	0.02
93	0.93	15	0.9206	0.03
93	0.93	16	0.9196	0.02
93	0.93	17	0.9208	0.04
93	0.93	18	0.9215	0.05
93	0.93	19	0.92211	0.07
93	0.93	20	0.9246	0.22

Figure S1: ROC curves for identifying CRO susceptibility using 8 machine learning models. (A) adaptive boosting (AB) model. (B) k-nearest neighbors (KNN) model. (C) light gradient boost (LGB) model. (D) logistic regression (LR) model. (E) Gaussian naive Bayes (GNB) model. (F) random forest (RF) model. (G) support vector machine (SVM) model. (H) extreme gradient boosting (XGB) model.

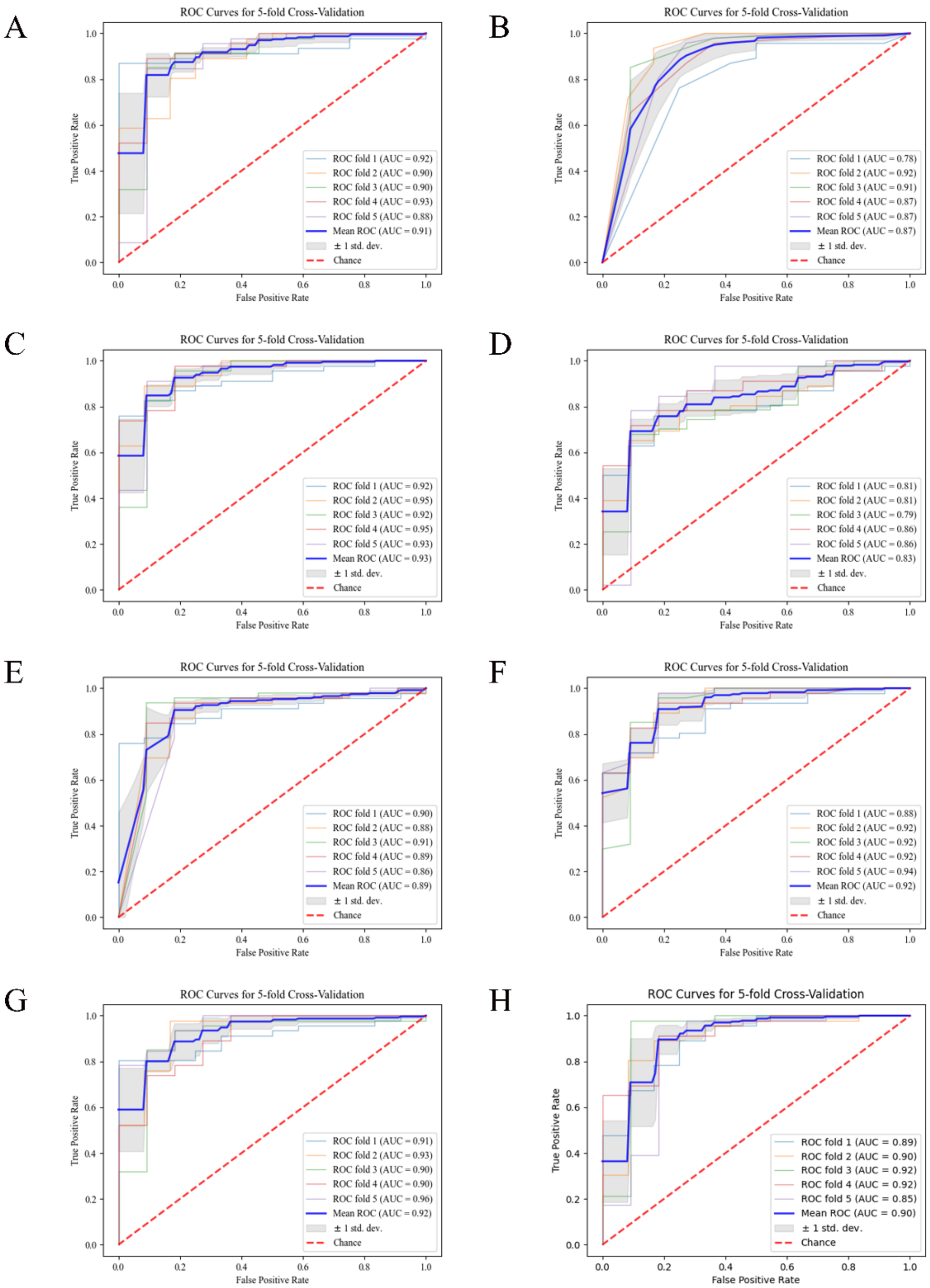

Figure S2: PR curves for predicting CRO susceptibility using eight machine learning models. (A) AB model. (B) KNN model. (C) LGB model. (D) LR model. (E) GNB model. (F) RF model. (G) SVM model. (H) XGB model.

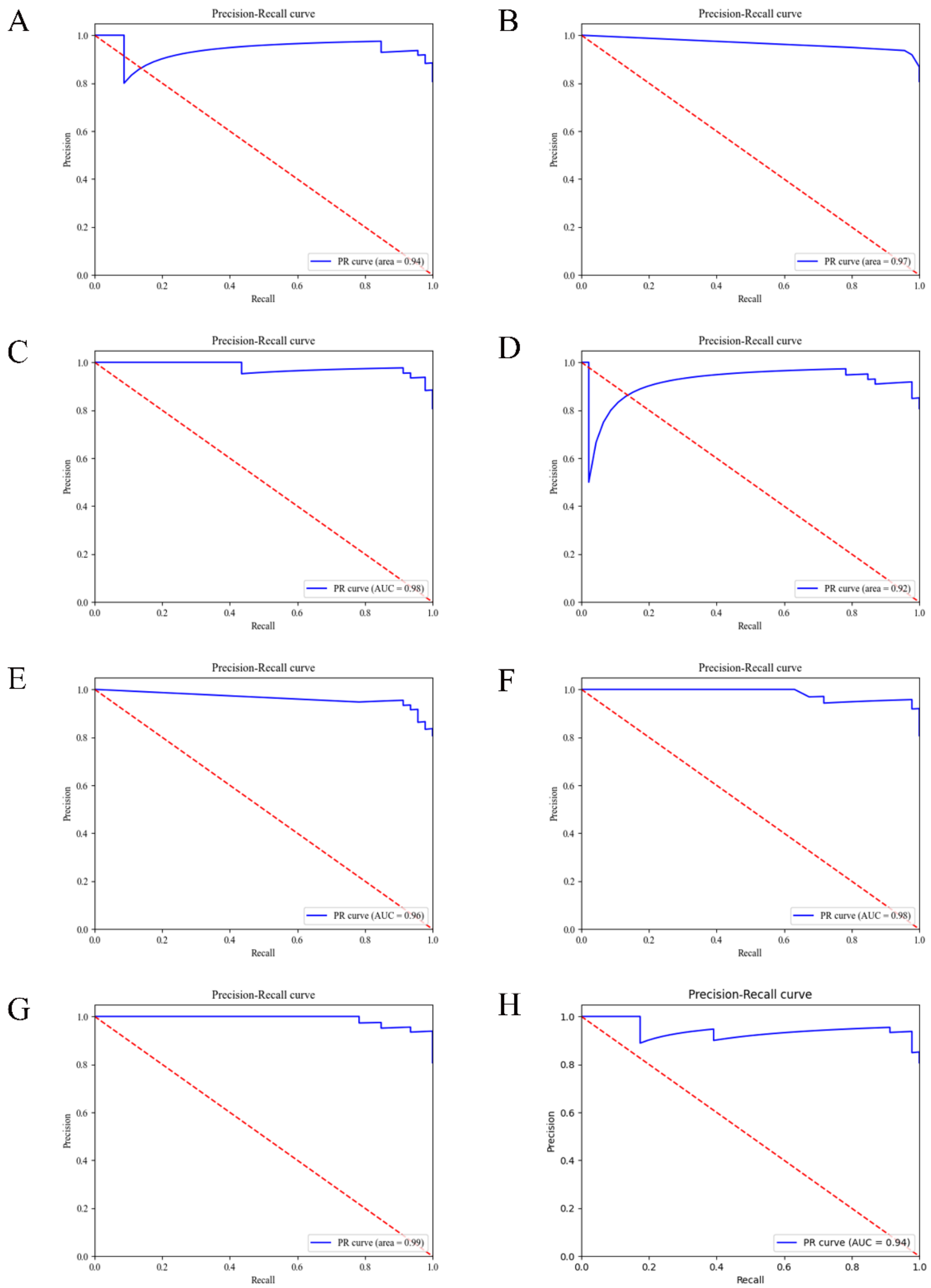

Figure S3: The ROC curves, PR curves and confusion matrix heat maps of the AB model with 7 features on the (A, E, I) training set, (B, F, J) internal validation set, external (C, G, K) WCGH set and (D, H, L) SUCH set.

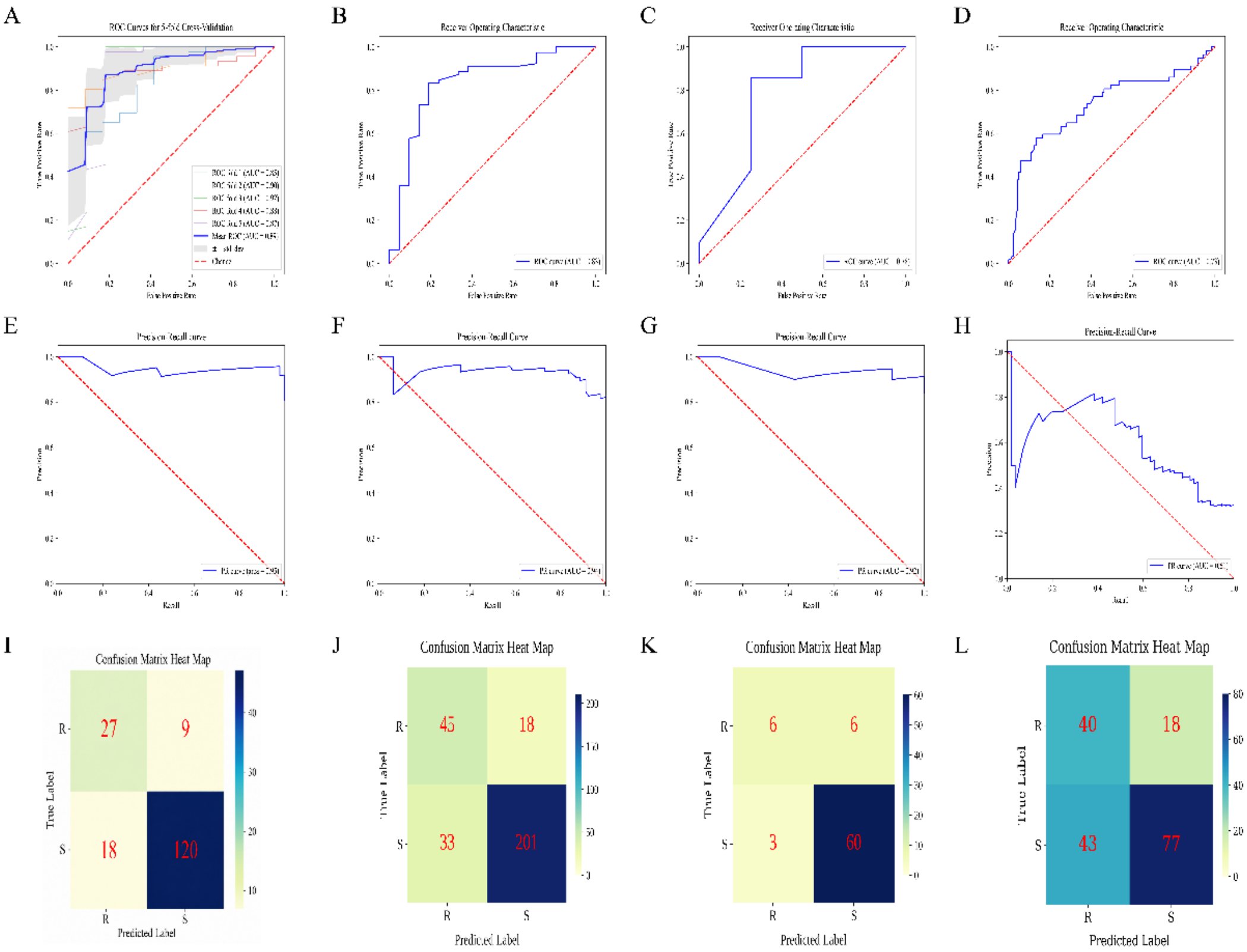

Figure S4: The ROC curves, PR curves and confusion matrix heat maps of the GNB model with 7 features on the (A, E, I) training set, (B, F, J) internal validation set, external (C, G, K) WCGH set and (D, H, L) SUCH set.

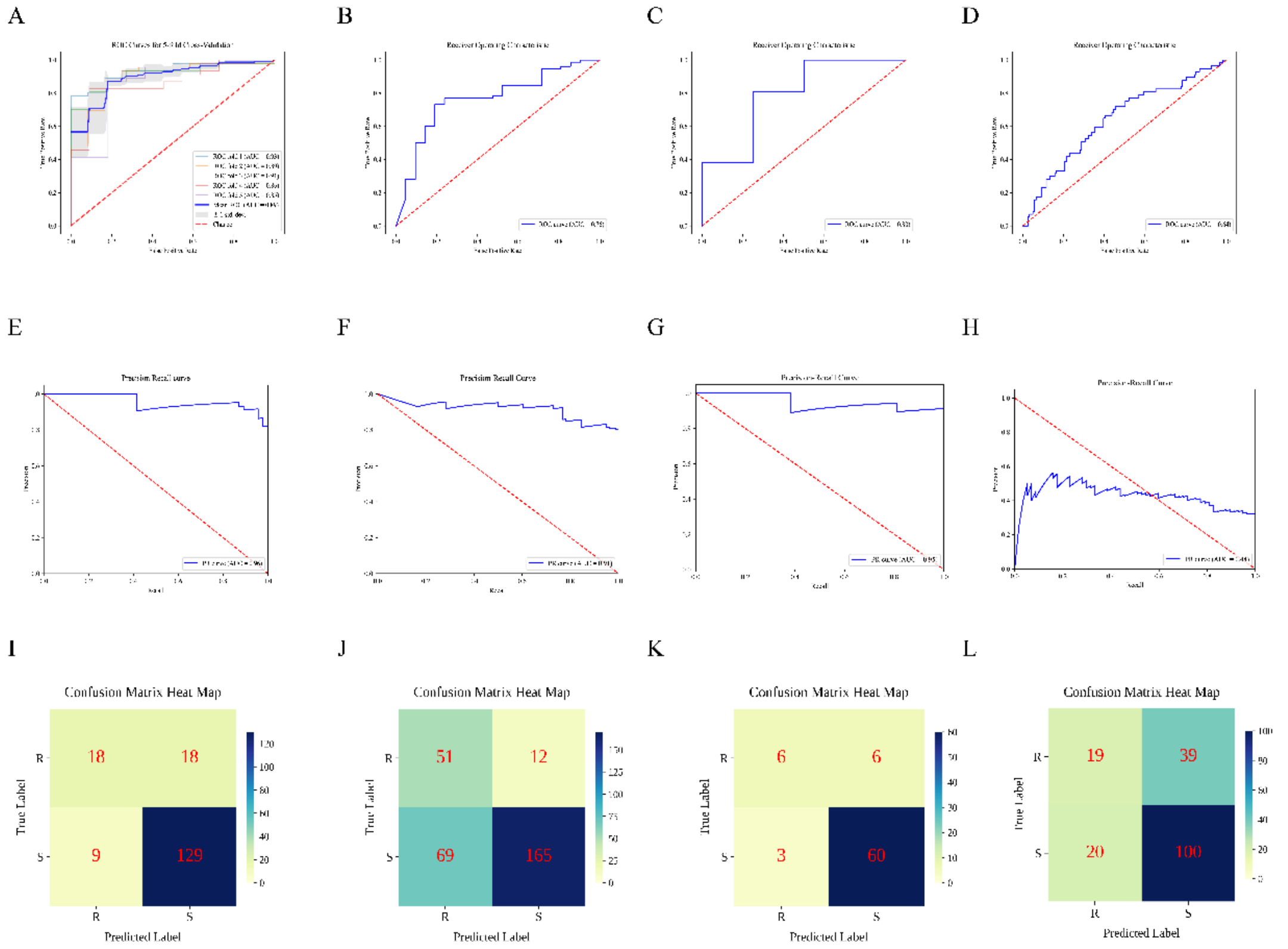

Figure S5: The ROC curves, PR curves and confusion matrix heat maps of the RF model with 7 features on the (A, E, I) training set, (B, F, J) internal validation set, external (C, G, K) WCGH set and (D, H, L) SUCH set.

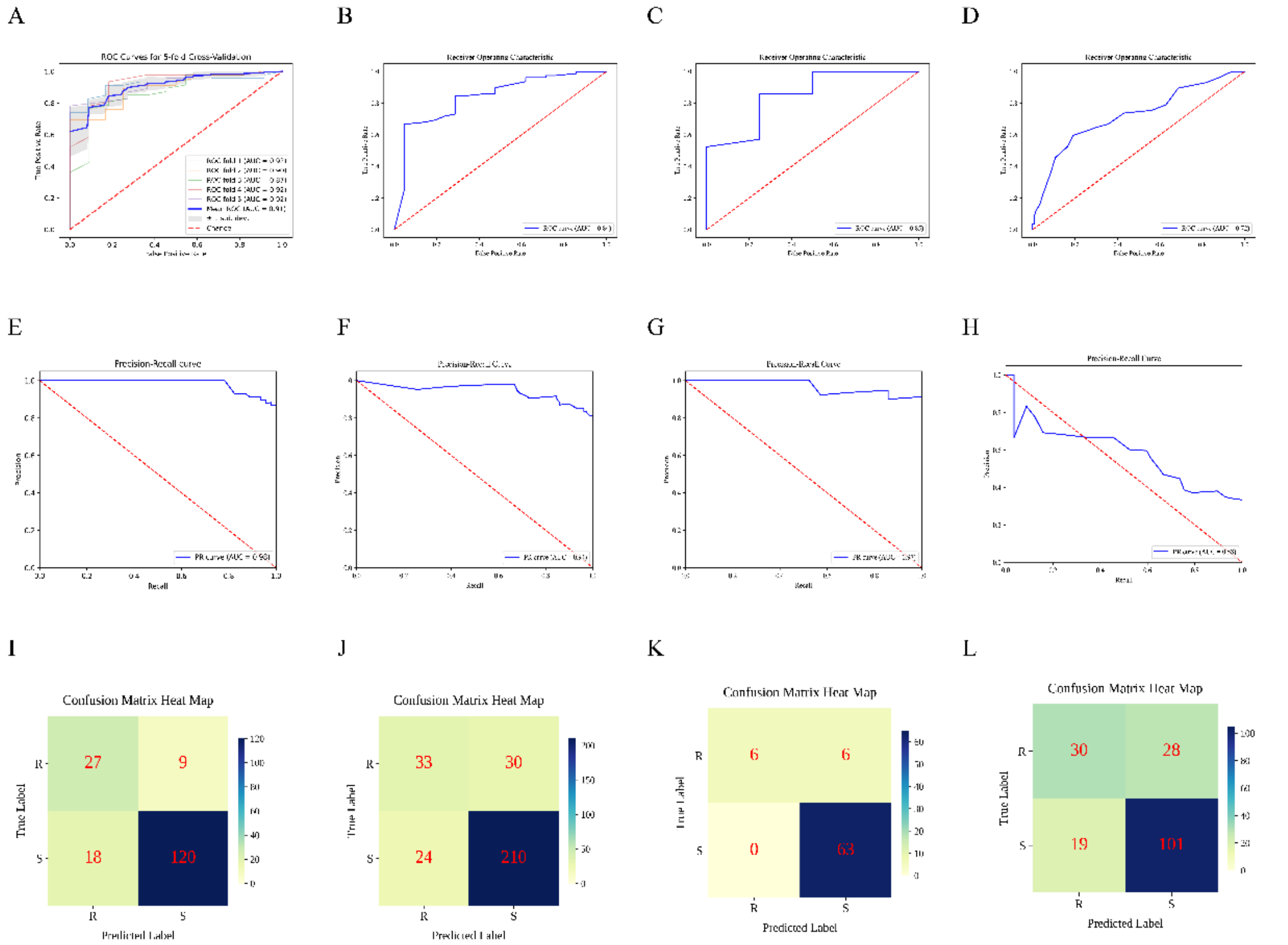

Figure S6: The ROC curves, PR curves and confusion matrix heat maps of the XGB model with 7 features on the (A, E, I) training set, (B, F, J) internal validation set, external (C, G, K) WCGH set and (D, H, L) SUCH set.

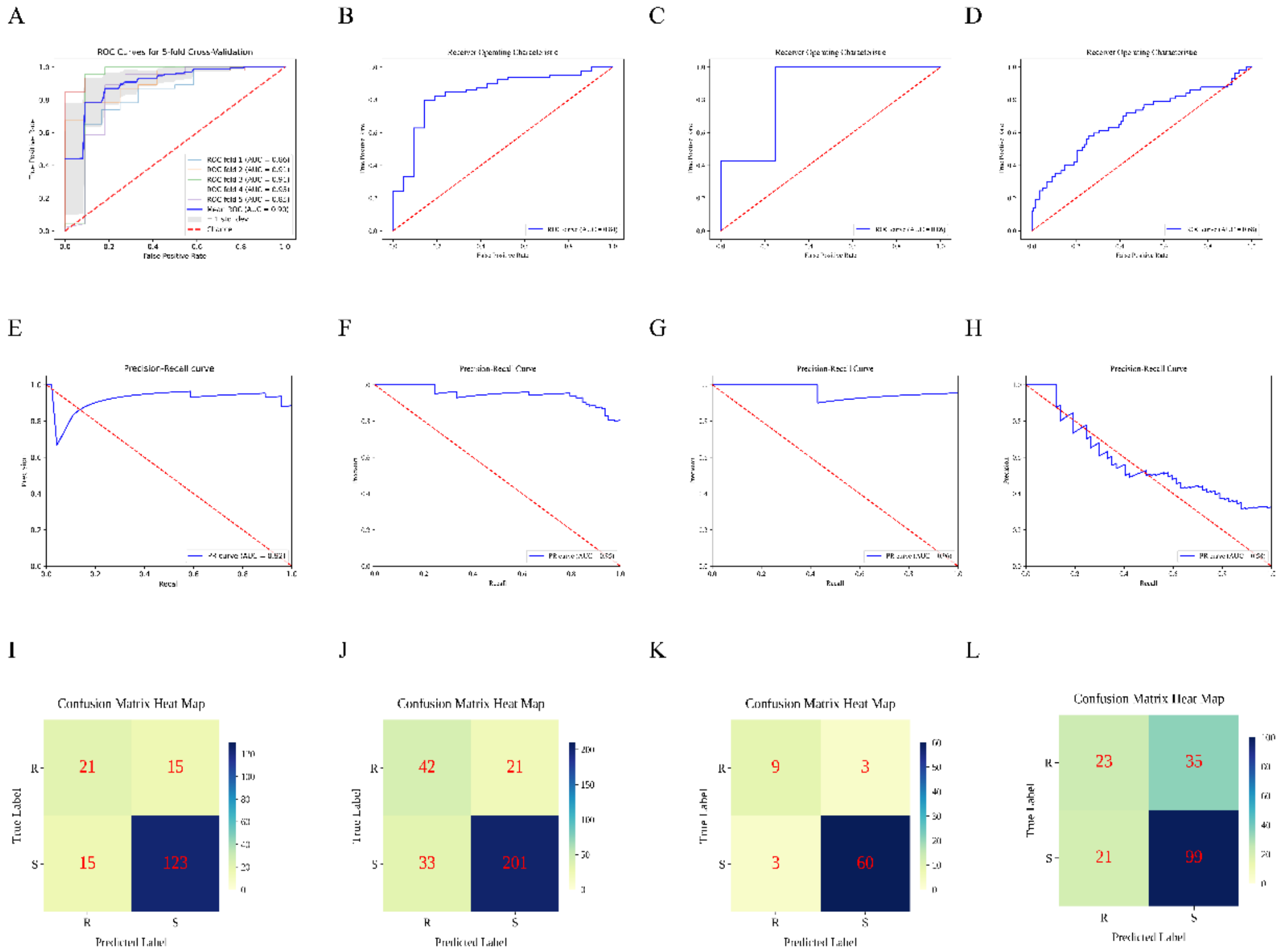

Figure S7: SHAP dependence and waterfall plots for interpretation of the final model. (A-G) SHAP dependence plots for the seven most influential features used by the final model. Each point represents one isolate in the dataset, with the x-axis showing the feature value and the y-axis showing the SHAP value, indicating the feature's impact on the model output. (H-I) SHAP waterfall plots for two representative NTS isolates, illustrating how individual features contribute to the predicted probability of ceftriaxone resistance. Red bars indicate features that increase the predicted risk, while blue bars indicate features that reduce it.

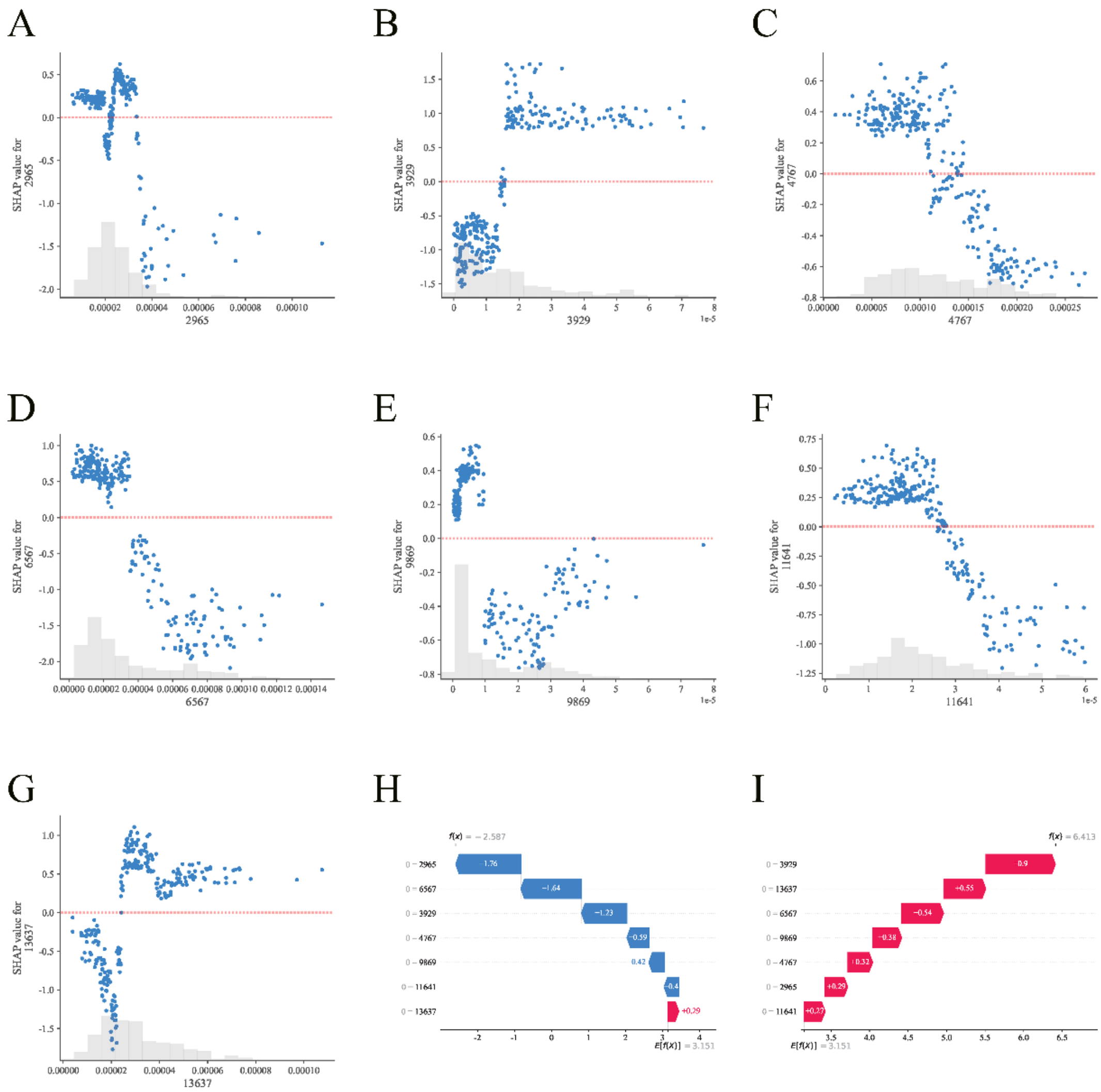

Figure S8: The PR curves of the final LGB model with 7 features on the internal and external validation sets. (A) Internal validation set. (B) External validation 1 (WCGH). (C) External validation 2 (SUCH).

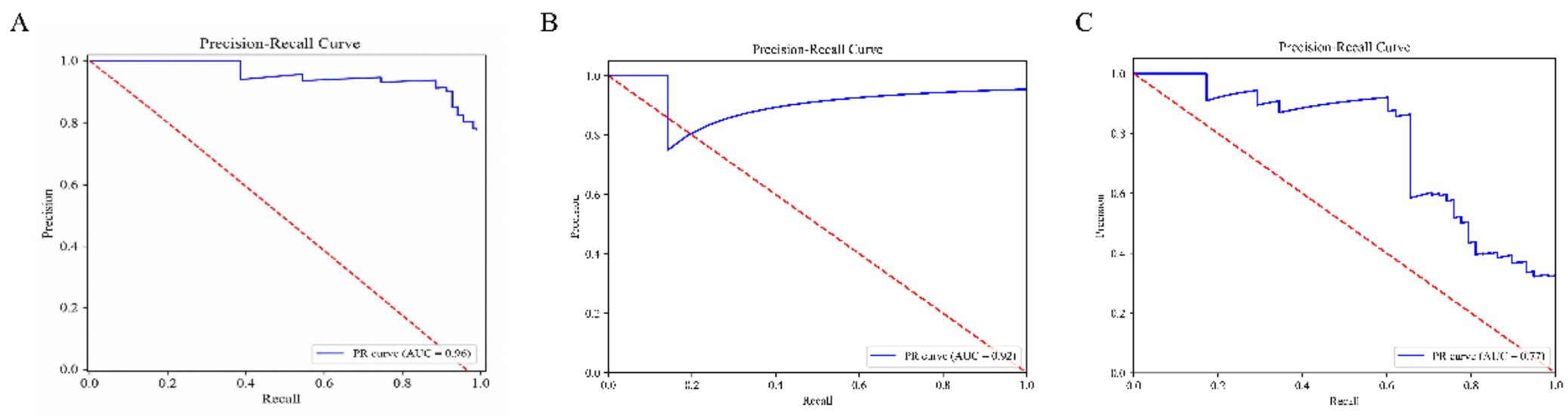

Figure S9. Decision curve analysis (DCA) of the LGB model in three datasets.
(A) Training set. (B) Internal validation set. (C) External validation 1 (WCGH). (D) External validation 2 (SUCH). The model demonstrated consistently higher net benefit across a range of threshold probabilities compared to the “treat-all” and “treat-none” strategies in all three datasets.

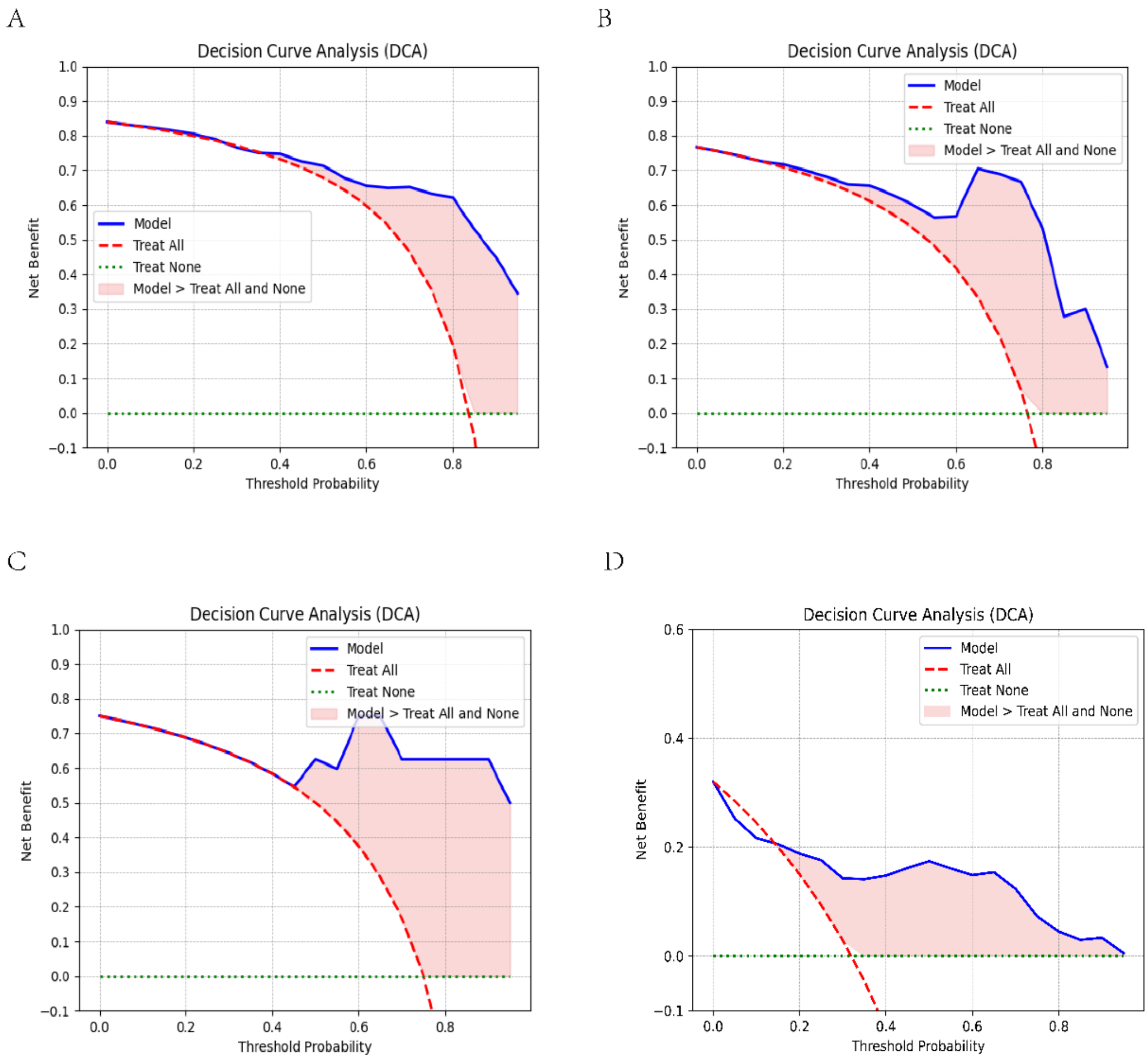

Figure S10: A convenient tool for laboratory use. A user-friendly application based on the final LGB model with 7 features is available for predicting CRO susceptibility in NTS. (A) Feature input interface. (B) Prediction results interface

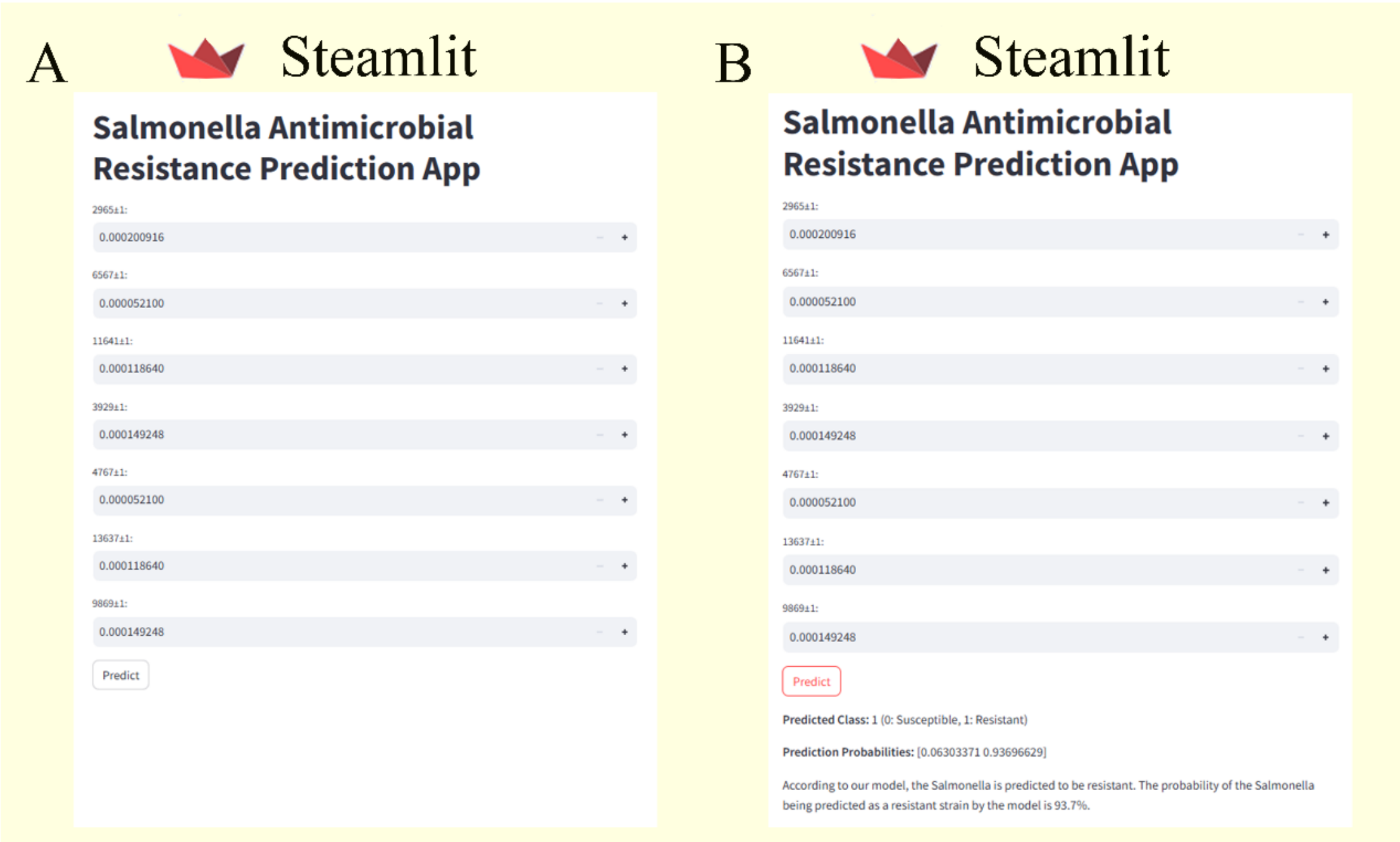

15. Code: Data processing of MALDI-TOF MS protein spectra

```
library('MALDIquant')
library('MALDIquantForeign')
library('readxl')

samples = read_excel('')
spectra = importTxt(getwd())
spectra = transformIntensity(spectra, method = "sqrt")
spectra = smoothIntensity(spectra, method = "SavitzkyGolay", halfWindowSize = #)
spectra = removeBaseline(spectra, method = "SNIP", iterations = #)
spectra = calibrateIntensity(spectra, method = "TIC")
avgSpectra = averageMassSpectra(
   spectra,
   labels = samples$group[match(
      samples$file,
      sapply(spectra, function(s) {
         tail(strsplit(s@metaData$file, '[\\/]')[[1]], 1)
      })
   )]
)
avgSpectra = alignSpectra(
   avgSpectra,
   halfWindowSize = #, SNR = #,
   tolerance = #, warpingMethod = "lowess"
)
peaks = detectPeaks(avgSpectra, method = "MAD", halfWindowSize = #, SNR = #)
peaks = binPeaks(peaks, tolerance = #)
intensity = intensityMatrix(peaks, avgSpectra)
intensity = t(intensity)
colnames(intensity) = samples$group[match(
   sapply(peaks, function(s) {
      tail(strsplit(s@metaData$file, '[\\/]')[[1]], 1)
   }),
   samples$file
)]
write.csv(intensity, '')
```

16. Supplementary Protocol: Step-by-step workflow for using the MALDI-TOF MS–based Streamlit prediction tool.

Colony selection:

Select a fresh, well-isolated nontyphoidal *Salmonella* colony after overnight culture. The isolate should first be confirmed as nontyphoidal *Salmonella* using routine laboratory identification and serological testing.

Accepted culture medium:

The current model was developed and validated only using isolates grown on Salmonella-Shigella agar. Spectra obtained from isolates grown on other media should not be used unless additional validation or model retraining has been performed.

MALDI-TOF MS sample preparation:

Apply a single colony to the MALDI target spot. Add 1 μL of 70% formic acid and allow the spot to dry at room temperature. Then add 1 μL of HCCA matrix solution and allow the spot to dry again.

Spectral acquisition:

Acquire spectra using the Bruker MALDI-TOF MS platform in the range of 2,000–20,000 Da. Each isolate should be spotted in triplicate. Spectra with identification scores below 2.0 should be excluded from further analysis.

Spectral preprocessing and feature extraction:

Export spectra in TXT format and process them using the preprocessing pipeline described in the Supplementary Code. The seven required m/z features are 2965, 6567, 11641, 3929, 4767, 13637, and 9869. The corresponding relative intensity values should be extracted after preprocessing.

Data entry into the Streamlit tool:

Open the web application and enter the relative intensity values of the seven required features into the corresponding input fields. Users should ensure that the units and preprocessing method are consistent with those described in this study. Missing, raw, unprocessed, or manually estimated peak values should not be entered.

Prediction and interpretation:

Click the “Predict” button after all seven values have been entered. The tool will display the predicted probabilities of ceftriaxone-susceptible and ceftriaxone-resistant outcomes. The category with the higher predicted probability is shown as the model prediction.

Reporting and clinical use:

The result should be interpreted as a preliminary prediction only. The current tool is not intended to replace conventional antimicrobial susceptibility testing. Final clinical reporting and antimicrobial treatment decisions should still be based on standard AST results and clinical judgment.
